# Prevalence and factors associated with mental distress among men at higher risk of HIV infection enrolled in an HIV pre-exposure prophylaxis program in Tanga, Tanzania

**DOI:** 10.3389/fpsyt.2026.1605734

**Published:** 2026-01-22

**Authors:** Nuruel Robert Kitomary, Emmy Metta, Melkizedeck T. Leshabari, Kåre Moen, Elia J. Mmbaga

**Affiliations:** 1 Department of Behavioural Sciences, Muhimbili University of Health and Allied Sciences, Dar Es Salaam, Tanzania; 2 Department of Community Medicine and Global Health, University of Oslo, Oslo, Norway; 3 Department of Epidemiology and Biostatistics, Muhimbili University of Health and Allied Sciences, Dar Es Salaam, Tanzania

**Keywords:** men who have sex with men, MSM, pre-exposure prophylaxis, PrEP, Tanzania

## Abstract

**Background:**

Data on the extent of mental health among men who have sex with men (MSM) in Tanzania are scarce, but studies elsewhere report a high prevalence of mental distress, mainly depression and anxiety symptoms. Mental distress has been linked to stigma, rejection, violence, and inadequate social support, with increased HIV vulnerability and poor uptake of preventive interventions such as pre-exposure prophylaxis (PrEP) and antiretroviral treatment. This study aimed to determine the prevalence of and factors associated with mental distress among men who have sex with men on PrEP in Tanga, Tanzania.

**Methods:**

This paper is based on data emanating from the control arm of a pragmatic quasi-experimental trial for HIV PrEP rollout in Tanzania with registration number MUHAS-REC-12-2024–2542 accordingly. The study population included men aged 18 years or older initiating PrEP. Participants were recruited through respondent-driven sampling (RDS). Mental distress was assessed at baseline using the Patient Health Questionnaire - two-question version (PHQ-2) and Generalized Anxiety Disorder - two-question version (GAD-2) for depressive and anxiety symptoms, respectively. A modified Poisson regression model was used to determine independent factors associated with mental distress.

**Results:**

Our study found that 16.8% of MSM on PrEP experienced mental distress. Factors associated with higher prevalence of mental distress included high self-perceived HIV risk (aPR=2.29, 95% CI = 1.14–4.59, p=0.020), low PrEP knowledge (aPR=2.7, 95% CI = 1.29–5.64, p=0.020), and high PrEP stigma (aPR=1.65, 95% CI = 1.03–2.64, p=0.036). Participants reporting to access condoms easily had a lower prevalence of mental distress (aPR=0.57, 95% CI = 0.35–0.94, p=0.028).

**Conclusions:**

Mental distress was relatively high among MSM, especially those with low PrEP knowledge, high PrEP stigma, and perceived high HIV risk, while easy condom access lowered the prevalence. Healthcare stakeholders should work to enhance PrEP awareness, reduce stigma, and ensure condom availability. Expanding and integrating mental health services within HIV programming is crucial in achieving the goal of ending the HIV epidemic by 2030.

## Introduction

Currently, approximately14.3% of the global population is experiencing a mental health condition with nearly two-third of the affected individuals living with depression, anxiety or both, according to the World Health Organization (1). In Tanzania, mental health conditions remains a public health problem with no specific world’s rank report but some population specific studies have reported a higher prevalence of depression (70%) five times than general population (2). As compared to the general population, men who have sex with men (MSM) present with higher rates of mental distress (3–5). Global data on mental distress among men in this population has focused on symptoms such as depression (6, 7), anxiety (8, 9), and stigma (10–12) and sleep disturbances (13) only. In the same context, few studies have provided data on the overall prevalence of mental distress as exemplified by an Indian study that shows 52.9% of MSM experience mental distress symptoms, nearly four times higher than the global average (14, 15). Depressive and anxiety symptoms are the main presentation of mental distress among MSM; for example, systematic reviews have shown that 35% of men who have sex with men have depressive symptoms (16), and 32.2% have anxiety symptoms (16). In East Africa countries, previous studies report depressive symptoms to be in the range of 15.8% to 16.1%, with anxiety symptoms at 12.7% in Kenya (17, 18) and 8.9% in Rwanda (19), among men who have sex with men, no study has combined depressive symptoms and anxiety symptoms. In Tanzania, a study by Mgopa and colleagues found depressive symptoms among MSM who use drugs to be as high as 70% (2). In Tanzania, having sex with another man is criminal, and punishable according to Tanzania penal code section 154 and 157, which create fear, hostile environment, with other factors such as low social support, stigma, violence, social rejection, substance use cause as copying strategy and possible anxiety and depressive symptoms (mental distress), as reported in previous studies (20–23).

In this context, this population remains at elevated risk of other health conditions including, communicable diseases such as human immunodefiency virus infections (HIV), risk that may be exacerbated by the dynamic mental health states that the experience. Human immunodeficiency virus (HIV) infections remain a major global health challenge, affecting approximately 39.9 million people worldwide, and are closely linked to mental distress (24). Key and vulnerable populations (KVP), including MSM, account for up to 21% of new HIV infections, with an increased risk of HIV acquisition up to twenty-three times compared to the general population (25). On average, MSM have been estimated to have an eleven-fold (7.7%) higher prevalence of HIV compared to heterosexual individuals (0.7%) worldwide (26). A study in Dar es Salaam, Tanzania, reported the prevalence of HIV among MSM (8.3%) to be double (27) that of the general population (4.8%) (28). Men who have sex with men are more vulnerable to HIV due to factors such as increased susceptibility of rectal mucosa to HIV due to anal sex, stable partners, unprotected sexual intercourse, multiple sexual partners, poor health-seeking behaviors, mental distress symptoms, and substance use comorbidities (29–31). These challenges not only increase their vulnerability to HIV but also heighten the risk of mental distress (depression and anxiety symptoms) with some maladaptive coping mechanisms like alcohol and drug use (32). The presence of mental distress symptoms has been attributed to inconsistent PrEP and antiretroviral treatment use and poor sexual health practices, further heightening the chances of new infections and undermining the effectiveness of HIV treatments and intervention prevention (33–39).

In Tanzania, PrEP was introduced in 2017 as part of a comprehensive guideline for the management of HIV among KVPs based on the WHO recommendations (40). Following the establishment of the guidelines and a set of criteria for PrEP use, including the age requirements of at least 15 years, the roll-out started in 2021 and is ongoing to date (40). During the demonstration trials, up to 97% of enrolees expressed willingness to take PrEP (13). However, during follow-up, retention in PrEP care was reported to be between 27.7% and 47%, which describes a wide discrepancy between willingness and actual behavior (41, 42). To achieve the 2030 goal of ending HIV as a public health challenge, studies focusing on ways of promoting HIV prevention among the MSM population in Tanzania, a population estimated to be about 49,700 in 2014 (43), are called for.

A study involving women at higher risk of HIV found positive mental distress (depressive and anxiety symptoms) to be associated with early disengagement from PrEP use in Dar es Salaam (44) calling for an evaluation of any similar link among MSM. Moreover, literature and clinical practice demonstrate the intrinsic relationship between mental distress and neglected self-care, low motivation, loss of hope, low self-esteem, substance use, and risky behaviors (45–47). However, existing studies have focused more on willingness and knowledge about PrEP, leaving gaps in understanding the mental health experiences of enrollees (27, 48, 49). No studies on mental distress among MSM on pre-exposure prophylaxis have been conducted in Tanzania. This study, therefore, addressed such a gap by determining the prevalence and factors associated with mental distress among men who have sex with men who are enrolled in HIV pre-exposure prophylaxis in Tanga, Tanzania.

## Methods

### Study design and setting

This study presents an analysis of data from the control group of a pragmatic quasi-experimental trial evaluating the rollout of PrEP in Tanzania, known as the PREPTA study. The trial focused on two key populations, which were men who have sex with men (MSM) and female sex workers (FSWs) across two regions: Dar Es Salaam (intervention group) from March 2021 to July 2022 and Tanga (control group) from February 2022 to June 2023. Although the PREPTA project has been presented in detail in our previous publications (41, 44, 50), the trial was designed to assess the use of mHealth App in promoting the adherence to HIV pre-exposure prophylaxis among men who have sex with men and female sex workers from coastal regions, Dar es salaam, a largest commercial city of Tanzania with over 5 million people as an intervention Arm; Tanga city among cities with high HIV prevalence in Tanzania with over 2 million as a control arm for the intervention. This paper is the first explicitly analyzing the data from the men who have sex with men in Tanga, representing real-world conditions of PrEP implementation in Tanzania. HIV prevalence among men who have sex with men in Tanga was reported at 11.1% in a study by Ross and colleagues ten years ago (20) making it one of the higher prevalence in Tanzania. This study was conducted at Ngamiani Health Center, a centrally located governmentally owned health facility in Tanga. The health center offers PrEP services through a dedicated building to ensure convenient and discreet access for key populations, including men who have sex with men. The center provides care and treatments (CTC) services including sexual and reproductive health education, antiretroviral medications and condoms to approximately … people annually. In the same clinic a HIV pre-exposures medications are provided to key and vulnerable population such as men who have sex with men.

### Study population and eligibility

The study involved men who had sex with men who were starting PrEP during the recruitment. Eligibility criteria included being aged at least 18 years of age, having had sex with a man in the last 3 months, and having lived in the city of Tanga for the past 6 months. Other inclusion criteria include creatinine clearance exceeding 60 mL/min and consent to initiate PrEP according to the national HIV guideline (51).

### Sample size

The sample size was calculated using a Cochrane formula a proportion of 50% because no previous retrievable studies have determined mental distress among men who have sex with men or other key and vulnerable populations in Tanzania. Therefore, we utilized the formula hereunder.


$$\text{n}=\frac{Z^{2}P\;(1-P)}{e^{2}}$$

Where n is the minimum sample size, Z (1.96) =z-score corresponding to the degree of confidence, P is an estimated proportion of mental distress (50%), and e (5%) =desired precision. After computation, the obtained minimum sample size was 384. However, this paper utilized already collected data; a cohort consisting of all 369 men who have sex with men in the control group in Tanga was included in the analysis. The sample size for the PREPTA study was estimated using a formula designed to calculate sample sizes in cohort studies, considering the RDS recruitment technique (52, 53). Moreover, the available sample size (369) differed by 15 subjects from the expected sample size (384); therefore, we calculated the margin of error to check the validity and ability of the sample size to conclude with accuracy. Therefore, we used the formula for an infinite population, which states that the margin of error (MOE) = $\frac{Z\surd P(1-P)}{n}$, $\frac{1.96\surd 0.5(1-0.5)}{384}$= 5.10%. This margin of error is statistically acceptable because it is almost ±5% (54, 55); hence, the available data were adequate to answer the research question.

### Recruitment procedures

During the HIV pre-exposure prophylaxis rollout in Tanzania (PREPTA), the recruitment procedure applied respondent-driven sampling (RDS). The process began with three initial participants, called “seeds.” These seeds were purposefully selected to ensure diversity in age, geographic location, and educational background. Peer educators assisted in identifying seeds with extensive social networks. Each seed received three unique invitation coupons to be used to invite their peers to take part in the study. These peers constituted the first wave of recruitment. Before leaving the study center, they were, in turn, invited to invite their peers, who came to form the second wave of recruitment, followed by subsequent waves until the target sample size had been achieved. Participants received education from the research team regarding PrEP, including its definition, purpose, benefits, and refill schedules. The data collection for this study was done during the parental trial, where the baseline data collection, included assessment of depressive symptoms, anxiety symptoms and socio-demographic characteristics and sexual characteristics.

### Measurements

#### Dependent variable

The primary outcome was mental distress. In this paper, mental distress was defined as a participant screening positive (score ≥3) for depressive symptoms using a Patient Health Questionnaire (PHQ-2) and or screening positive (score ≥3) for anxiety symptoms using the Generalized Anxiety Disorder (GAD-2). The PHQ-2 assesses anhedonia, i.e., how often the patient has had “little interest or pleasure in doing things” in the past two weeks, and depressed mood, i.e., “feeling down, depressed, or hopeless. Items are responded to on a Likert-type scale from 0 (Not at all) to 3 (Nearly every day) for a total score that ranges between 0 and 6 (56, 57). Similarly, the GAD-2 assesses “Feeling nervous, anxious, or on edge” and “Not being able to stop or control worrying” for the past two weeks. Items are responded to on a Likert-type scale from 0 (Not at all) to 3 (Nearly every day) for a total score that can range between 0 and 6 (56, 57).

Both PHQ-2 and GAD-2 tools were translated to Swahili language, they are short versions and good for quick community-based population screening. They have been validated in Tanzania with good internal consistency at Cronbach alpha of 0.81 by Materu and colleagues (58, 59).

#### Independent variables

The independent variables in the study were grouped into socio-demographic characteristics which included the following, age group (18–24 years and ≥25 years), education level (grouped as primary education or low and secondary education or higher), marital status (categorized as never married versus ever married), having own children (classified as either being a father to a child or never having had a child), easy access to condoms (i.e., whether one can get a condom timely when one wants to have sex either dispensed in hotels, in health facilities or can buy), financial dependents (whether or not the participant had people who directly depended on him to get money), monthly income (all income he collected via formal or informal employment in a month), history of facing financial difficulties to accessing healthcare (inability to accessing healthcare due to lack of money to pay for that service), history of sexually transmitted diseases in last six months (any history of sexually transmitted disease based of different symptoms like urethral discharge), HIV knowledge (knowing HIV transmission and protective factors from five questions such as “either mosquito can transmit HIV or no”, “having one-HIV negative partner as health sexual behavior in preventing HIV transmission” etc. which was later categorized as yes or no), twelve-month history of forced sex (being engaged in anal sex without his willingness), self-reported HIV status (yes versus no), PrEP self-efficacy (extent of his belief that PrEP can offer protection against HIV transmission), prior arrests by police. Social support was measured using an 8-item, 5-point Likert scale adapted from the Duke-UNC Functional Social Support Questionnaire (FSSQ), which demonstrated high reliability with a Cronbach’s alpha of 0.88. A total score below 32 indicated inadequate social support (41, 50). Alcohol use was assessed using the Alcohol Use Disorder Identification Test (AUDIT), where a score of ≤ 7 indicated low risk, 8–15 reflected hazardous/harmful use, and ≥ 16 identified as alcohol dependence/alcohol use disorder (60). Perceived stigma against men who have sex with men was evaluated using 13 items, each with five response options (1 = strongly disagree to 5 = strongly agree), yielding a high reliability with a Cronbach’s alpha of 0.84. Moreover, stigma was categorized into low (scores ≤ 26), moderate (scores 27–38), and high (scores ≥ 39). Furthermore, perceived PrEP stigma was measured using a 10-item scale with five response options, achieving a Cronbach’s alpha of 0.88. Lastly, the PrEP stigma was classified as low (scores ≤ 30) or high (scores >30); all these were composed in the baseline questionnaire (61). These questionnaires were translated into Swahili language which is national language and well-spoken in the country including Tanga region the study site, all were pre-tested before actual data collection took place.

### Data analysis

Descriptive statistics for continuous variables were summarized using means and standard deviations. In contrast, categorical variables, such as education level, were presented as proportions and differences between proportions were assessed using the chi-square test. In the bivariate analyses, factors that were found to be associated with mental distress with a p-value <0.2 and those identified in previous literature as relevant to the outcomes were included in the regression analysis. Given that the prevalence of mental distress (16.80%) exceeds 10%, the modified Poisson regression with robust standard errors was used to determine independent factors associated with mental distress and respective adjusted prevalence ratios are reported. This method was chosen because it minimizes the risk of overestimating effects, which can occur when using a conventional logistic regression model (62). Variables with a p-value <0.05 in the multivariable modified Poisson regression model were considered statistically significant. All analyses were conducted using STATA, version 18.

## Results

### Distribution of socio-demographic characteristics by mental distress among men who have sex with men

The mean (±SD) age of the study participants was 24.7 (±5.5) years. More than half (58.80%) of the participants were in the age category between 18 and 24 years. Most had never been married (87.50%), and nearly two-thirds (65.80%) reported having no children. More than two-thirds (67.20%) had a secondary level of education or higher. Two-thirds (65.60%) of participants had financial dependents, and half (50.25%) reported having experienced financial difficulties due to health expenses. More than three-quarters (76.40%) had inadequate social support, and almost half (48.63%) had alcohol dependence (**Table 1**).

**Table 1:** Distribution of sociodemographic characteristics by mental distress among MSM on HIV pre-exposure prophylaxis in Tanga, Tanzania.

Variable	Total N (%),	Mental distress		*P-value*
		No n (%)	Yes n (%)	
Age groups (years) (24.7 ± 5.5)				0.207
18-24	217 (58.82)	185 (85.25)	32 (14.75)	
25+	152 (41.23)	122 (80.26)	30 (19.74)	
Marital status				0.338
Never married	323 (87.50)	271 (83.90)	52 (16.10)	
Ever married	46 (12.50)	36 (78.26)	10 (21.74)	
Has own children				0.603
Yes	126 (34.20)	103 (81.75)	23 (18.25)	
No	242 (65.80)	203 (83.88)	39 (16.12)	
Level of education				0.402
Primary and below	121 (32.80)	105 (84.00)	16 (16.00)	
Secondary and above	248 (67.21)	202 (81.45)	46 (18.55)	
Financial dependents				0.328
Yes	242 (65.61)	198 (81.82)	44 (18.18)	
No	127 (34.40)	109 (85.83)	18 (14.17)	
Total income per month (shillings)				0.284
<=100,000	116 (31.70)	95 (81.90)	21 (18.10)	
100,001-299,999	137 (37.40)	110 (80.29)	27 (19.71)	
>=300,000	113 (30.90)	99 (87.61)	14 (12.39)	
History of having faced financial difficulties due to health spending				0.040
Yes	184 (50.27)	146 (79.35)	38 (20.65)	
No	182 (49.73)	159 (87.36)	23 (12.64)	

### The distribution of structural and sexual behavior characteristics by mental distress

The mean age at sexual debut among the participants was 15.9 years, with a standard deviation of 2.7 (µ ± SD: 15.9 ± 2.7). Nearly three-quarters (70.73%) had their sexual debut before 18, with over a third (37.10%) having engaged in sex that involved anal, oral, or thigh contact. Over a quarter (29.27%) had engaged in anal sex before the age of 18, and more than two-thirds (68%) had had a female as their first sexual partner. Most (71.00%) preferred the insertive position in anal sex, with 77.78% taking that position in their most recent anal sex. Over half (51.2.0%) had a steady partner, while 55.86% had multiple partners. More than two-thirds (69.29%) had not used a condom during their last anal sex, but 77.11% had used lubricants at the last anal sex. Three-quarters (75.00%) had ever paid for sex, and a large majority (88.85%) had never engaged in group sex. Among those who had paid for sex, 73.17% had not used a condom during their last anal sex. Over a third (37.70%) reported having ever experienced forced sex in the past year, and 15.40% had been arrested over the past 12 months due to their sexual orientation. Nearly two-thirds (65.00%) had been tested for HIV, and 77.60% had easy condom access. Almost three-quarters (73.68%) perceived themselves to be at high risk for HIV, while 70.20% had no comprehensive HIV knowledge. Around 35.00% faced moderate sexual orientation stigma, and 14.10% experienced high sexual orientation stigma. Regarding PrEP, 66.40% had low knowledge, and 33.60% reported stigma, while 72.60% of them had high PrEP self-efficacy. Among all factors, preferred position in anal sex (p=0.049), self-perceived HIV risk (p=0.037), comprehensive HIV knowledge (p=0.022), and PrEP knowledge (p=0.001) were significantly associated with mental distress. In contrast, other factors were not (**Table 2**).

**Table 2:** Distributions of structural and sexual behaviors by mental distress among MSM on Pre-exposure prophylaxis program in Tanga, Tanzania.

Variable	Total N (%)	Mental distress		*P-value*
		No n (%)	Yes n (%)	
Age (in years) at sexual debut (15.9 ± 2.7)				0.511
<18	261 (70.73)	215 (82.38)	46 (17.62)	
18+	108 (29.27)	92 (85.19)	16 (14.81)	
Type of sex at sexual debut				0996
Anal/Oral/Thigh	137 (37.10)	114 (83.21)	23 (16.79)	
Vaginal	232 (62.90)	193 (83.19)	39 (16.81)	
Age at first anal sex (18.7 ± 4.5)				0.784
<18	149 (29.27)	123 (82.55)	26 (17.45)	
18+	220 (70.73)	184 (83.64)	36 (16.36)	
First sex partner				0.726
Male	118 (32.00)	97 (82.20)	21 (17.80)	
Female	251 (68.00)	210 (83.67)	41 (16.33)	
Anal sex position				0.049
Insertive	262 (71.00)	216 (82.44)	46 (17.56)	
Receptive	32 (08.70)	23 (71.88)	09 (28.12)	
Versatile	75 (20.30)	68 (90.67)	07 (09.33)	
Last anal sex position				0.544
Insertive	287 (77.78)	216 (82.44)	46 (17.56)	
Receptive/Versatile	82 (22.22)	91 (85.05)	16 (14.95)	
Steady male partner				0.060
Yes	189 (51.20)	164 (86.77)	25 (13.23)	
No	180 (48.80)	143 (79.44)	37 (20.56)	
Multiple sex partners				0.385
Yes	205 (55.86)	174 (84.88)	31 (15.12)	
No	162 (44.14)	132 (81.48)	30 (18.52)	
Using a condom in the last anal sex				0.538
Yes	113 (30.71)	96 (84.96)	17 (15.04)	
No	255 (69.29)	210 (82.35)	45 (17.65)	
History of using any lubricant				
Yes	283 (77.11)	232 (81.98)	51 (18.02)	
No	85 (22.89)	74 (87.06)	11 (12.94)	
Frequency of lubricant use				0.546
Never used	85 (23.10)	74 (87.06)	11 (12.94)	
Sometimes	90 (24.50)	74 (82.22)	16 (17.78)	
Always	193(52.40)	158 (81.87)	35 (18.13)	
Used lubricant last anal sex				0.304
Never used	85 (23.10)	74 (87.06)	11 (12.94)	
Yes	241 (65.50)	200 (82.99)	41 (17.01)	
No	42 (11.40)	32 (76.19)	10 (23.81)	
Ever paid for oral or anal sex				0.602
Yes	276 (75.00)	228 (82.61)	48 (17.39)	
No	93 (25.00)	79 (84.95)	14 (15.05)	
History of engaging in group sex				0.688
Yes	41 (11.14)	35 (85.37)	06 (14.63)	
No	327 (88.85)	271 (82.87)	56 (17.13)	
Used condom last time paid someone to have anal or oral sex				0.145
Yes	99 (26.83)	87 (87.88)	12 (12.12)	
No	270 (73.17)	220 (81.48)	50 (18.52)	
Forced sex lasts 12 months				0.105
Yes	139 (37.70)	110 (79.14)	299 (20.86)	
No	230 (62.30)	197 (85.65)	33 (14.35)	
Arrested last 12 months due to sexual orientation				0.584
Yes	57 (15.40)	46 (80.70)	11 (19.30)	
No	312 (84.60)	261 (83.65)	51 (16.35)	
Ever tested for HIV				0.699
Yes	240 (65.00)	201 (83.75)	39 (16.25)	
No	129 (35.00)	106 (82.17)	23 (17.83)	
Can easily access male condoms when in need				0.060
Yes	284 (77.60)	243 (85.56)	41 (14.44)	
No	82 (22.40)	63 (76.83)	19 (23.17)	
Diagnosed with STIs in the past 6 months				0.502
Yes	44 (11.99)	35 (79.55)	09 (20.45)	
No	323 (88.01)	270 (83.59)	53 (16.41)	
Self-perceived HIV risk				0.037
High risk	266 (73.68)	213 (80.08)	53 (19.92)	
Medium risk	33 (09.14)	30 (90.91)	03 (09.09)	
Low/no risk	62 (17.17)	57 (91.94)	05 (08.06)	
Comprehensive HIV knowledge				0.022
No	259 (70.20)	223 (86.10)	36 (13.90)	
Yes	110 (29.80)	84 (76.36)	26 (23.64)	
Perceived stigma against men who have sex with men				0.780
Low	188 (50.90)	155 (82.45)	33 (17.55)	
Moderate	129 (35.00)	107 (82.95)	22 (17.05)	
High	52 (14.10)	45 (86.54)	07 (13.46)	
PrEP knowledge				0.001
Low	245 (66.40)	193 (78.78)	52 (21.22)	
High	124 (33.60)	114 (91.94)	10 (08.06)	
PrEP stigma				0.062
Low	294 (79.70)	250 (85.03)	44 (14.97)	
High	75 (33.60)	57 (76.00)	18 (24.00)	
PrEP self-efficacy				0.215
Low (<24 = 24)	101 (27.40)	88 (87.13)	13 (12.87)	
High (>24)	268 (72.60)	219 (81.72)	49 (18.28)	
Level of social support				0.839
Inadequate	282 (76.40)	234 (82.98)	48 (17.02)	
Adequate	87 (23.60)	73 (83.91)	14 (16.09)	
Alcohol use				0.452
Low risk	150 (41.21)	129 (86.00)	21 (14.00)	
Harmful or hazardous use	37 (10.16)	31 (83.78)	06 (16.22)	
High risk	177 (48.63)	143 (80.79)	34 (19.21)	

### Factors associated with mental distress

Participants who self-perceived their HIV risk to be high had a prevalence of mental distress that was more than double that of those with low self-perceived HIV risk (aPR=2.29, 95% CI = 1.14–4.59, p=0.020). Participants with low PrEP knowledge had nearly three times higher prevalence of mental distress (aPR=2.7, 95% CI = 1.29–5.64, p=0.020) than those with high PrEP knowledge. Those experiencing high levels of PrEP stigma had 1.65 the prevalence of mental distress compared to those with low PrEP stigma (aPR=1.65, 95% CI = 1.03–2.64, p=0.036). Conversely, participants who reported easy access to condoms had a significantly lower prevalence of mental distress (aPR=0.57, 95% CI = 0.35–0.94, p=0.028) compared to those who reported difficulty accessing condoms (**Table 3**).

**Table 3:** Modified Poisson regression analysis for factors associated with mental distress among men who have sex with men. .

Variables	Crude estimates of the prevalence ratio		Adjusted estimates of prevalence ratio	
	cPR (95%CI)	*P-value*	aPR (95%CI)	*P-value*
Age groups (years)				
18-24	Ref.		Ref.	
25+	1.34 (0.85-2.11)	0.208	1.31 (0.766-2.24)	0.324
Level of education				
Primary and below	Ref		Ref.	
Secondary and above	1.40 (0.83-2.37)	0.208	1.46 (0.91-2.34)	0.117
Total income per month				
<=100,000	1.46 (0.78-2.73)	0.235	1.36 (0.69-2.66)	0.372
100,001-299,999	1.59 (0.88-2.89)	0.127	1.59 (0.87-2.90)	0.132
>=300,000	Ref		Ref.	
Multiple Sex Partners				
Yes	1.13 (0.71-1.81)	0.336	0.71 (0.45-1.10)	0.115
No	Ref		Ref.	
Age at first anal sex				
<18	1.07 (0.67-1.69)	0.784	0.97 (0.52-1.83)	0.936
18+	Ref		Ref.	
Type of sex at sex debut				
Anal/Oral/Thigh	1.00 (0.62-1.60)	0.996	0.89 (0.50-1.60)	0.697
Vaginal	Ref		Ref.	
Last Anal Sex Position				
Insertive	Ref		Ref.	
Receptive/Versatile	0.93 (0.53-1.63)	0.796	1.06 (0.62-1.81)	0.822
Ever paid for having oral or anal sex				
Yes	1.16 (0.67-2.00)	0.606	1.00 (0.57-1.75)	0.999
No	Ref		Ref.	
Forced sex lasts 12 months				
Yes	1.45 (0.92-2.29)	0.105	1.28 (0.78-2.12)	0.329
No	Ref		Ref.	
Arrested last 12 months due to their sexual orientation				
Yes	1.18 (0.66-2.13)	0.580	0.94 (0.51-1.71)	0.836
No	Ref		Ref.	
Ever tested for HIV				
Yes	0.91 (0.57-1.46)	0.699	0.79 (0.50-1.25)	0.314
No	Ref		Ref.	
Can easily access male condoms when in need				
Yes	0.58 (0.37-0.93)	0.024	0.57 (0.35-0.94)	**0.028**
No	Ref			
Diagnosed with STIs in the past 6 months				
Yes	1.25 (0.67-2.36)	0.483	1.22 (0.62-2.41)	0.557
No	Ref		Ref.	
Self-perceived HIV risk				
High risk	2.28 (1.17-4.46)	0.016	2.29 (1.14-4.59)	**0.020**
No risk	Ref		Ref.	
Experienced stigma				
<=26 (Low)	Ref		Ref.	
27-38 (Moderate)	0.97 (0.59-1.59)	0.908	0.98 (0.60-1.60)	0.943
>=39(High)	0.77 (0.36-1.63)	0.492	0.94 (0.44-1.99)	0.873
Comprehensive HIV knowledge				
No	0.59 (0.37-0.93)	0.022	1.54 (0.96-2.50)	0.054
Yes	Ref		Ref.	
PrEP knowledge				
Low	2.63 (1.38-5.00)	0.003	2.70 (1.29-5.64)	**0.008**
High	Ref		Ref.	
PrEP stigma				
Low	Ref		Ref.	
High	1.60 (0.99-2.61)	0.057	1.65 (1.03-2.64)	**0.036**
PrEP self-efficacy				
Low (<24 = 24)	0.70 (0.40-1.24)	0.226	1.32 (0.70-2.47)	0.391
High (>24)	Ref		Ref.	
Level of social support				
Inadequate	1.06 (0.61-1.82)	0.840	0.83 (0.49-1.43)	0.511
Adequate	Ref		Ref.	
Alcohol use				
Low risk	Ref		Ref.	
Moderate risk	1.16 (0.50-2.67)	0.730	0.82 (0.36-1.90)	0.660
High-risk use	1.37 (0.83-2.26)	0.214	1.13 (0.66-1.20)	0.682

The bolded numbers in the last column of the table indicate a statistically significant variable

## Discussion

We estimated the prevalence of mental distress and its associated factors among men who have sex with men (MSM) using HIV pre-exposure prophylaxis (PrEP) in Tanga, Tanzania. Our findings revealed that 16.8% of MSM on PrEP experienced mental distress among 367 participants. Of the total sample, 15.7% reported depressive symptoms and 10.7% experienced anxiety symptoms. The prevalence of mental distress was higher among participants who perceived themselves to be at high risk for HIV, who had lower PrEP knowledge, and who faced significant PrEP-related stigma. Conversely, those who reported easy access to condoms exhibited a lower prevalence of mental distress than those who reported difficulty accessing condoms.

The overall prevalence of mental distress in our study (16.8%), this prevalence was relatively higher compared to that reported in other studies in Africa among men who have sex with men, ranging between 7.5% and 12.5% in Nigeria and Togo, respectively (17, 63). Also, in contrast to our study, the Indian study reported a prevalence of 52.9% of different mental distress among men who have sex with men (14). This difference could partly be attributed to the broader scope of the previous study and the nature of participants that a study from Nigeria and Togo involved other KVPs; even if they were able to give specific prevalence in MSM, others screened for all symptoms of mental disorders. Furthermore, our study focused solely on depressive and anxiety symptoms and specifically targeted MSM who were enrolled in PrEP services. The differences in assessment tools (17, 63) could also play a role. A range of social, cultural, political, and genetic differences (14) could also have played a role in this difference.

The prevalence of depressive symptoms in our study (15.7%) was relatively low compared to estimates from a prior systematic review of studies among men who have sex with men, which reported a prevalence ranging from 7% to 71% across various nations (64). This variation may reflect differences in MSM population characteristics, including social, cultural, legal, and genetic differences, the magnitude of HIV epidemics, stigma and legal matters around homosexuality from different countries. For instance, a study by Li and colleagues in China, which focused on MSM living with HIV, reported a 55.8% prevalence of depressive symptoms (9). Similarly, a study by Mgopa and colleagues in Tanzania found a 70% prevalence of depressive symptoms among men who have sex with men who are using different substances (19). The differences in these two studies compared to our research would possibly be related to substance withdrawal dysphoria (sadness, loss of interest, low energy, low motivation, and generalized body weakness) among drug users (65, 66) or HIV-related depressive disorders among MSM living with HIV (45, 67, 68) which led them to use drugs as self-medication by using various substances such as alcohol and heroin (47, 69). Despite the lower prevalence of depressive symptoms in our study, the rate was significant, approximately four times higher than that of the men’s general population (4%) and even that of the uncategorized general population (3.8%) (70). The four times higher prevalence of depression warrants the need for tailored screening and interventions for the possible depressive symptoms among men who have sex with men. Poor mental health among members of this population has synergistically been linked to increased HIV transmission and reduced uptake of both preventive and care services (71–73). This is because depression presents with low motivation, neglected self-care, poor health care, increased high risk activities, hopelessness, and sometimes experiences of violence.

Anxiety symptoms, a component of mental distress, were reported by 10.7% of participants in our study. This is relatively low compared to the estimated pooled prevalence of anxiety among MSM (32.2%), which ranges from 12.7% to 57.6% across different nations (16). The differences may be due to variations in population characteristics, as some studies focused on MSM living with HIV (9, 74), and others were meta-analyses and systematic reviews (74). The relatively low levels of anxiety in our study population may be attributed to their exposure to HIV-related knowledge and PrEP counseling, which could alleviate fears related to HIV acquisition (75–77). This group may have benefited from prior involvement in education interventions on prevention methods and multiple counseling sessions (77). The high prevalence of anxiety among MSM in this population highlights the need for special attention to mental health in this population, this could be linked fear of rejection, stigma and insecurity due to their sexual orientation. This findings underscores the urgent need to integrate routine anxiety screening and sexual minority affirming mental health support into PrEP services, as unaddressed anxiety driven by minority stress may undermine sustained engagement and retention in HIV prevention care services.

Our study found that participants who reported easy access to condoms had a 43% lower prevalence of mental distress compared to those who did not have easy access to condoms. This association between condom access and mental distress has been well-established in previous research. For example, a study by Hill and colleagues in Tanzania found that high levels of anxiety were prevalent among individuals who reported not using condoms (46). Our findings are also consistent with studies that show individuals struggling with their mental health, such as fear and adjustments to the possibility of getting HIV, are more likely to engage in risky sexual behaviors (78). This aligns with the health risk perception theory, which suggests that individuals’ emotional and psychological reactions are influenced by their perceptions of health risks, such as the possibility of HIV infection after poor protection due to poor condom access (79). Improving access to condoms presents a big opportunity in reducing mental distress, promoting good healthcare services utilization and consequently might play a role in reducing the HIV transmission rate among at-risk populations.

MSM who perceived themselves to be at high risk of HIV in this study had twice the prevalence of mental distress compared to those who did not. The relationship between the self-perceived HIV risk and mental health has been reported in a study by Niag in Malawi (80). Our findings are also in line with a study conducted in China by Dan Wu colleagues, which found that MSM who identified HIV as a risk to their lives were more likely to have depression compared to their counterparts (8). The link between mental distress and perceived HIV risk is explained by health risk theory, which posits that perceiving oneself at high risk heightens subjective fear and worry especially after engaging in risky behaviors, such as unprotected sex (79). Perceiving a higher risk of HIV plays a two-sided sword role that, while it is important to take precautions, individuals who perceive themselves to be at a higher risk of contracting HIV may experience mental distress. This distress may stem from fear and the need for adjustment, particularly if individuals have engaged in risky sexual behaviors and fear being diagnosed with HIV. These feelings are often shaped by subjective judgments about the likelihood of contracting HIV, which can lead to negative emotions (81). These findings show that although perceiving oneself at higher risk of HIV can encourage protective behaviors, it can also bring fear, worry, and emotional disturbances. PrEP programs should therefore be both risk-focused messaging and empathetic, client-cantered health education with integrated mental health support, helping individuals acknowledge their fears, adapt emotionally, and remain engaged in HIV prevention care.

Furthermore, this study found that men who have sex with men with low knowledge about HIV pre-exposure prophylaxis had nearly three times higher prevalence of mental distress compared to those with high knowledge. Studies are limited regarding the relationship between PrEP knowledge and mental distress. However, few studies have looked at the link between HIV knowledge and the use of preventive measures, such as the use of HIV pre-exposure prophylaxis (82). Our study findings are in line with the study in China, which found that MSM who had little knowledge about HIV had a higher prevalence of both depressive and anxiety symptoms compared to their counterparts (8). A possible explanation is the heightened perception of HIV acquisition risk (79), driven by repeated exposure to potentially risky sexual behaviors and the uncertainty surrounding the outcomes of these encounters. This uncertainty may stem from not fully understanding the level of risk involved or being unaware of appropriate protective measures to take after engaging in such behaviors, such as using post-exposure prophylaxis (PEP) following unprotected sexual intercourse (40). Continued reproductive health education targeting HIV prevention and treatments presents the potential opportunity to reducing mental distress by doughty clearing, shared experience by peer led groups and hence improve emotional wellbeing.

This study also found that men who have sex with men who reported to have experienced high levels of PrEP-related stigma had nearly twice the prevalence of mental distress compared to those with low levels of stigma. Our study findings are consistent with the findings of a community-based PrEP clinic study in Guatemala, which reported a higher prevalence of depressive symptoms among PrEP users (83). Qualitative studies have identified stigma as a structural factor influencing HIV PrEP use, where PrEP-related stigma has emerged as an important theme in these findings, with PrEP often being linked to promiscuity and mistreatment (10, 81). Additionally, systematic reviews have shown that people living with HIV with a high stigma level are more likely to experience depressive or anxiety symptoms compared to those with low stigma levels (20). This is consistent with our findings underscoring the mechanisms and consequences of stigma (social isolation, discrimination, violence, shame, and fear) as the cause of mental distress (2, 14). While the World Health Organization, scientists, and public health teams are advocating PrEP use in breaking HIV transmission among men who have sex with men, an accompanying effort should focus on community awareness, which will help with PrEP normalization and, hence, less stigma and mental distress. Although mental health services are available at all health facilities from the health center level in Tanzania, they operate as separate units. Integrating routine mental health screening and treatment into HIV Care and Treatment Clinics (CTC) is feasible through the regular presence of trained mental health personnel within these clinics.

### Ethical consideration

The study was reviewed and received ethical clearance registration number MUHAS-REC-12-2024–2542 from the Muhimbili University of Health and Allied Sciences Ethical Review Committee in Tanzania. The study project from which data for this paper emanate received ethical approval from the Regional Committee for Medical and Health Research Ethics (REK) in Norway (protocol ref no: 33675). The study was conducted while observing all local legislation and institutional requirements. The participants provided their written informed consent to participate in this study.

### Study limitation

The study had several limitations. Participants were recruited based on their eligibility for PrEP; hence, they may not be representative of the MSM population. The use of respondent-driven sampling methods, a non-probabilistic method in the recruitment of respondent-driven sampling methods, and a non-probabilistic method in the recruitment may have introduced selection bias. However, regression analysis of RDS data has been indicated to be accurate in estimating predictors of outcome similar to estimates from probabilistic sampling. However, this paper analyzed secondary data which exhibited missing data less than 5% across a few variables that are not critical to the findings. Participants who screened positive for symptoms of depression and anxiety did not undergo further diagnostic evaluation or referral to a psychiatrist or clinical psychologist, as the study was not designed to provide clinical diagnosis or treatment, but an important consideration for the future studies. Finally, the study relied on reported behaviors, and given the nature of this study, social desirability bias may have affected our estimates. However, the use of standardized measures and efforts to ensure confidentiality and anonymity might have addressed the potential bias.

## Conclusions and recommendations

The prevalence of mental distress was relatively high among men who had sex with men in this study. MSM with low PrEP knowledge, high PrEP-related stigma, and a heightened perception of HIV risk had a higher prevalence of mental distress, while those reporting easy access to condoms had a lower prevalence. The findings highlight the need for healthcare stakeholders to contribute to enhancing PrEP awareness campaigns, promote HIV education, and combat stigma while ensuring widespread condom availability. This can be achieved by lodging the routine mental health screening within CTC and PrEP services, task-shifting basic mental health care to trained HIV service providers, strengthening referral linkages with mental health units, and ensuring continuous capacity building and supportive supervision for healthcare workers.

## Data Availability

The raw data supporting the conclusions of this article will be made available by the authors, without undue reservation.
